# Supplementary material for: The Impact of COVID-19 on the Service of Emergency Department
Source: Healthcare (Basel). 2021 Sep 29;9(10):1295. doi: 10.3390/healthcare9101295 (PMC8544500; doi:10.3390/healthcare9101295)
Supplement: Supplementary file 1 [file healthcare-09-01295-s001.zip › healthcare-1333409-supplementary.pdf]

Supplementary Material: Table S1. Triage observation checklist

	Patient information					Assessor work experience	Arrival - triage to examination time			Delay	Notes
1	Age<br>…<br>…	Gender<br>☐ F<br>☐ M	Social status<br>☐ M<br>☐ S<br>☐ D	Patient nationality<br>☐ Saudi<br>☐ Non	Resident place [In Taif]<br>☐ Yes<br>☐ No	Assessor experience<br>☐ <5<br>☐ 5 to 10<br>☐ >10	Patient arrival time:	Patient triaged time:	Patient seen by physician time:	☐ Yes<br>☐ No	If yes, why:
2	Age<br>…<br>…	Gender<br>☐ F<br>☐ M	Social status<br>☐ M<br>☐ S<br>☐ D	Patient nationality<br>☐ Saudi<br>☐ Non	Resident place [In Taif]<br>☐ Yes<br>☐ No	Assessor experience<br>☐ <5<br>☐ 5 to 10<br>☐ >10	Patient arrival time:	Patient triaged time:	Physicians' examination time:	☐ Yes<br>☐ No	If yes, why:
3	Age<br>…<br>…	Gender<br>☐ F<br>☐ M	Social status<br>☐ M<br>☐ S<br>☐ D	Patient nationality<br>☐ Saudi<br>☐ Non	Resident place [In Taif]<br>☐ Yes<br>☐ No	Assessor experience<br>☐ <5<br>☐ 5 to 10<br>☐ >10	Patient arrival time:	Patient triaged time:	Physicians' examination time:	☐ Yes<br>☐ No	If yes, why:
4	Age<br>…<br>…	Gender<br>☐ F<br>☐ M	Social status<br>☐ M<br>☐ S<br>☐ D	Patient nationality<br>☐ Saudi<br>☐ Non	Resident place [In Taif]<br>☐ Yes<br>☐ No	Assessor experience<br>☐ <5<br>☐ 5 to 10<br>☐ >10	Patient arrival time:	Patient triaged time:	Physicians' examination time:	☐ Yes<br>☐ No	If yes, why:
5	Age<br>…<br>…	Gender<br>☐ F<br>☐ M	Social status<br>☐ M<br>☐ S<br>☐ D	Patient nationality<br>☐ Saudi<br>☐ Non	Resident place [In Taif]<br>☐ Yes<br>☐ No	Assessor experience<br>☐ <5<br>☐ 5 to 10<br>☐ >10	Patient arrival time:	Patient triaged time:	Physicians' examination time:	☐ Yes<br>☐ No	If yes, why:
6	Age<br>…<br>…	Gender<br>☐ F<br>☐ M	Social status<br>☐ M<br>☐ S<br>☐ D	Patient nationality<br>☐ Saudi<br>☐ Non	Resident place [In Taif]<br>☐ Yes<br>☐ No	Assessor experience<br>☐ <5<br>☐ 5 to 10<br>☐ >10	Patient arrival time:	Patient triaged time:	Physicians' examination time:	☐ Yes<br>☐ No	If yes, why:
7	Age<br>…<br>…	Gender<br>☐ F<br>☐ M	Social status<br>☐ M<br>☐ S<br>☐ D	Patient nationality<br>☐ Saudi<br>☐ Non	Resident place [In Taif]<br>☐ Yes<br>☐ No	Assessor experience<br>☐ <5<br>☐ 5 to 10<br>☐ >10	Patient arrival time:	Patient triaged time:	Physicians' examination time:	☐ Yes<br>☐ No	If yes, why:
8	Age<br>…<br>…	Gender<br>☐ F<br>☐ M	Social status<br>☐ M<br>☐ S<br>☐ D	Patient nationality<br>☐ Saudi<br>☐ Non	Resident place [In Taif]<br>☐ Yes<br>☐ No	Assessor experience<br>☐ <5<br>☐ 5 to 10<br>☐ >10	Patient arrival time:	Patient triaged time:	Physicians' examination time:	☐ Yes<br>☐ No	If yes, why:
